# Parenchymatous Mastitis Causing Disease in Humans

**Published:** 1896-04

**Authors:** 


PARENCHYMATOUS MASTITIS AS CAUSE OF ACUTE GASTRO-INTESTINAL CATARRH IN THE HUMAN BODY.[2] The streptococcus so commonly recognized as a disease-excitant in the human body has been found in recent times to be present in certain forms of diarrhœa. The writer had the opportunity in 1894, in Christiana, to investigate four different groups of acute gastro-intestinal catarrh, the cause of which he attributed to this micro-organism.

In the first group, eight individuals in two different families of the same street fell suddenly ill four hours after having par-

[1, 2] Translated for the Journal by Frank H. Miller, V.S., Royal Veterinary University, Berlin, Germany.

taken of milk. Those of the families who had either not used the milk or had partaken of it in a boiled state remained healthy, with the exception of one child, which had taken boiled milk and sickened, but only slightly. The appearance of the milk showed nothing abnormal, but it coagulated on boiling and showed large numbers of micrococci, particularly streptococci, indistinguishable from streptococcus pyogenes longus. This discovery led to the suspicion that the milk had been contaminated by the admixture of milk from cattle suffering from udder-inflammation of suppurative character. This was substantiated by Veterinarian Kolderup, who found a cow suffering from parenchymatous mastitis and concomitant diarrhœa. The milk of this animal, which had been diseased for about two weeks had been withheld from the rest until the day in question, when, a new attendant came to the dairy.

The second group, consisting of five persons, was attacked with acute gastro-enteritis a couple of hours after partaking of unboiled milk, and still later more suffered. In this case also Kolderup found a cow suffering from parenchymatous mastitis, and the milk entered the general supply in the manner recorded in the first group (by changing attendant). The general macroscopical appearance of the milk was normal, but it had numerous pus-cells which *contained* streptococci.

The third group was a mother and her child, who suffered in a similar manner from the use of "raw" milk, which upon examination showed numerous diplo- and streptococci. It was thin and flocculent and contained several small clots of pus; also among the supplying herd were found two suffering from mastitis parenchymatose.

The fourth group comprised four children of the same family, who were attacked after using new milk which to all macroscopical appearance appeared healthy. Kolderup found that upon the day of their attack a cow suffering from parenchymatous mastitis had been sold from the herd whence the milk came. The attendant was sick and another person had carried out the work, and it is fair to suppose the milk of the animal was mixed with that of the healthy animals.

The writer admits his inability to carry out an examination of the excreta of any of the patients, leaving a decided gap in the evidence, but feels that the foregoing facts establish a close relation between the character of the disease and the nature of the milk-supply.—HOLST, *Trans. Zeitschrift for Meat and Milk Hygiene, Berlin.*

